# Higher-order QCD predictions for dark matter production at the LHC in simplified models with *s*-channel mediators

**DOI:** 10.1140/epjc/s10052-015-3700-6

**Published:** 2015-10-07

**Authors:** Mihailo Backović, Michael Krämer, Fabio Maltoni, Antony Martini, Kentarou Mawatari, Mathieu Pellen

**Affiliations:** Centre for Cosmology, Particle Physics and Phenomenology (CP3), Université catholique de Louvain, 1348 Louvain-la-Neuve, Belgium; Institute for Theoretical Particle Physics and Cosmology, RWTH Aachen University, 52056 Aachen, Germany; Theoretische Natuurkunde and IIHE/ELEM, Vrije Universiteit Brussel, and International Solvay Institutes, Pleinlaan 2, 1050 Brussels, Belgium

## Abstract

Weakly interacting dark matter particles can be pair-produced at colliders and detected through signatures featuring missing energy in association with either QCD/EW radiation or heavy quarks. In order to constrain the mass and the couplings to standard model particles, accurate and precise predictions for production cross sections and distributions are of prime importance. In this work, we consider various simplified models with *s*-channel mediators. We implement such models in the FeynRules/MadGraph5_aMC@NLO framework, which allows to include higher-order QCD corrections in realistic simulations and to study their effect systematically. As a first phenomenological application, we present predictions for dark matter production in association with jets and with a top-quark pair at the LHC, at next-to-leading order accuracy in QCD, including matching/merging to parton showers. Our study shows that higher-order QCD corrections to dark matter production via *s*-channel mediators have a significant impact not only on total production rates, but also on shapes of distributions. We also show that the inclusion of next-to-leading order effects results in a sizeable reduction of the theoretical uncertainties.

## Introduction

Various astrophysical and cosmological observations provide strong hints for the existence of dark matter (DM). Yet, very little is known about the nature of DM, and constraints on models from various direct/indirect detection experiments and cosmology still allow for a wide range of DM masses and couplings to the Standard Model (SM) particles. Various types of DM searches are sensitive to different regions of the DM model parameter space [1, 2]. In order to maximise the chances for discovering – or at least excluding – DM models, it is hence imperative to perform both astrophysical and collider searches for DM. The most promising way to detect signals of weakly interacting DM particles at the LHC is through their associated production with jets, EW bosons and heavy quarks, leading to signatures with missing transverse energy (MET). Searches for DM have been performed at LHC Run I (see e.g. Refs. [3, 4]) and are one of the central goals of LHC Run II [5].

While the complementarity of different DM searches is a powerful tool, it is intrinsically limited in that the comparison of collider and other results introduces some degree of model dependence. As the nature of DM is still unknown, there exist a myriad of DM models and mechanisms to be explored, spanning a wide range of complexity and ambition. In this context, it is of utmost importance to follow an approach where the model dependence is limited, while the salient features that can provide a useful characterisation of possible signals are kept intact, i.e., the “simplified models” approach [6].

Many simplified models for DM have been proposed in the past (see Refs. [5, 7, 8] and references therein). In their simplest form, these models assume DM to be a single massive particle which interacts weakly with the SM particles. The interaction of DM with the SM can be mediated by a new field, which we dub the mediator. When the mediator is heavier than the energy scales the experiment can probe, the interaction becomes point-like and the Lagrangian can be organised in terms of a tower of higher-dimensional operators in the framework of an effective field theory (EFT). However, when the experimentally accessible energy becomes comparable or higher than the mediator mass, on-shell effects become important and a properly defined quantum field theory featuring the mediator state in the spectrum is needed [9–14]. The LHC can explore a large range of DM and mediator masses, as well as coupling strengths and possible combinations of DM and mediator spins. Collider results within the framework of simplified models can then be combined with direct searches for the mediators, e.g. in Drell–Yan processes, as well as with cosmological and astrophysical constraints on DM.

LHC searches for DM will rely on precision calculations to impose the most stringent bounds on DM models, and hopefully characterise possible signals. Higher-order corrections in QCD to DM production processes at the LHC are hence vital for extracting precise information about DM from the LHC results.

Previous analyses [15–19] studied next-to-leading order (NLO) QCD corrections for DM production in MET $+j/\gamma /W$ in the case of EFT, i.e., in the limit of heavy mediators.^1^ In this article, we consider simplified models with *s*-channel mediators. We analyse the impact of the higher-order corrections to mono-jet signals in various benchmark scenarios with spin-1 (vector and axial-vector) mediators, calculate DM production cross sections (both total and differential) at NLO accuracy with up to two jets, and also merge the corresponding samples via the FxFx procedure [20]. To our knowledge, such accuracy has so far not been achieved in the context of DM simulations/predictions for the LHC Run II.

In addition, we consider $t\bar{t}$ associated production and compute NLO cross sections and distributions for spin-0 (scalar and pseudo-scalar) mediators in representative cases, including those with a light mediator. Predictions for this class of processes at NLO in QCD also represent a novelty in the context of DM computations for LHC Run II.

The first goal of this work is to illustrate the feasibility of having a fully general implementation of DM simplified models in the FeynRules [21]/MadGraph5_aMC@NLO [22] (MG5aMC henceforth) framework, accurate up to NLO in QCD. To this aim we start with the simplest (yet non-trivial) case of *s*-channel mediators (colour singlet, spin 0 and 1 bosons) coupling to DM and quarks. We assume that DM is a Dirac fermion for concreteness, yet our implementation is not limited by the choice of DM spin or nature (Dirac or Majorana). We show how predictions and event generation for this class of models can be achieved at NLO QCD accuracy, *in a fully automatic way*, for a wide set of observable/final states, while also employing the most recent matching/merging multi-jet techniques [20].

The second goal of this work is of phenomenological nature, i.e. to investigate the impact of the NLO corrections on the current and future searches for DM at the LHC. We consider two examples, among several promising final state signatures:

1 $$\begin{aligned} pp \rightarrow X \bar{X} + \mathrm{jets} \end{aligned}$$

for a spin-1 mediator model, and

2 $$\begin{aligned} pp \rightarrow X \bar{X} + t \bar{t} \end{aligned}$$

for a spin-0 mediator model, where *X* is a DM particle. We do not only calculate NLO QCD corrections to the overall production rates, but also study in detail the effects of higher-order corrections on the differential distributions of relevant observables.

Our simulation set-up is based on the FeynRules/MG5aMC framework. The FeynRules package provides the relevant Feynman rules starting from any local Lagrangian [21, 23, 24], as well as the UV/$R_2$ counterterms [25] necessary for the NLO computations via Nloct [26]/FeynArts [27]. Our simplified DM model files are publicly available at the FeynRules repository [28]. With these ingredients, which are only based on the model and are not process dependent, MG5aMC computes tree-level amplitudes, loop-amplitudes [29–31] and subtraction terms for a desired process, as well as their integration over phase space [32]. Event generation is obtained by matching short-distance events to the parton shower employing the MC@NLO method [33], which is implemented for Pythia6 [34], Pythia8 [35], Herwig6 [36] and Herwig++ [37]. We note that our DM model files can be exported not only to event generators but also to tools for DM relic abundance as well as DM direct and indirect detections such as MicrOMEGAs [38, 39] and MadDM [40, 41], allowing for more comprehensive DM studies.

The paper is organised as follows. In Sect. 2 we introduce simplified models for DM and specify the relevant interactions and model parameters of the implementation. We discuss the impact of the NLO QCD corrections on DM pair production with jets through spin-1 mediators in Sect. 3, which includes a discussion of inclusive cross sections, differential distributions as well as a discussion of the impact of parton showers and the NLO merging of events with different jet multiplicities. NLO QCD predictions for DM production in association with a top-quark pair through spin-0 mediators are discussed in Sect. 4. We provide our conclusions and an outlook in Sect. 5.

## Simplified dark matter models: the *s*-channel mediator case

We start by defining the particle content and the interactions of the simplified model, which we dub DMsimp. We assume that DM is described by a single, massive and weakly interacting particle, that communicates with the SM through the exchange of a mediator. For simplicity, we assume that the mediator is not part of the SM.^2^ The first very general classification stems from the class of vertices that characterise the model: Lagrangians featuring DM–DM-mediator and SM–SM-mediator type interactions identify models with an *s*-channel mediator, while Lagrangians characterised by DM–SM-mediator interactions define a *t*-channel mediator. The former interactions, for example, arise in models featuring an extra scalar or $Z'$ which couples to a pair of DM particles, while the latter is common in supersymmetric models. From the point of view of QCD corrections the two classes are very different, as an *s*-channel mediator is necessarily a colour singlet, while a *t*-channel mediator can be either neutral or coloured. In this work we focus on the *s*-channel models, leaving the implementation and validation of *t*-channel models to forthcoming work. The *s*- and *t*-channel classes can be further organised by the quantum numbers of the DM particle and the mediator. To start with, we focus on the case of Dirac DM with spin-1 or spin-0 mediators coupling to the matter fields of the SM. Changing the spin or the nature of the fermion (Dirac or Majorana) of the DM particle or including a coupling of the mediator to the SM bosons is straightforward [42]. On the other hand, extending our analysis to spin-2 mediators, while feasible in principle, entails dedicated validation work, as such models are, in general, not renormalisable. We defer such an extension to the future.

### Spin-1 mediator model

In the framework of our simplified model, the interaction Lagrangian of a spin-1 mediator ($Y_1$) with a Dirac fermion DM ($X_\mathrm{D}$) is given by

3 $$\begin{aligned} \mathcal{L}_{X_\mathrm{D}}^{Y_1} = \bar{X}_\mathrm{D} \gamma _{\mu } (g^{\mathrm {V}}_{X_\mathrm{D}} +g^{\mathrm {A}}_{X_\mathrm{D}}\gamma _5)X_\mathrm{D}\,Y_1^{\mu }, \end{aligned}$$

and with quarks by

4 $$\begin{aligned} \mathcal{L}_\mathrm{SM}^{Y_1}&= \sum _{i,j} [\bar{d}_i\gamma _{\mu } (g^{\mathrm {V}}_{d_{ij}}+g^{\mathrm {A}}_{d_{ij}}\gamma _5)d_j \nonumber \\ {}&\quad +\bar{u}_i\gamma _{\mu } (g^{\mathrm {V}}_{u_{ij}}+g^{\mathrm {A}}_{u_{ij}}\gamma _5)u_j] Y_1^{\mu }, \end{aligned}$$

where *d* and *u* denote down- and up-type quarks, respectively, ($i,j = 1,2,3$) are flavour indices, and $g^{\mathrm{V}/\mathrm{A}}$ are the vector/axial-vector couplings of DM and quarks. Note that we adopt this notation according to the actual implementation in FeynRules. The model file, including an alternative choice for the spin of DM particle (complex scalar $X_\mathrm{C}$), can be downloaded at the FeynRules repository [28].

The pure vector and pure axial-vector mediator scenarios are given by setting the parameters in the Lagrangians (3) and (4) to

5 $$\begin{aligned}&g^\mathrm{V}_{X_\mathrm{D}} \equiv g_X \quad \mathrm{and}\quad g^\mathrm{A}_{X_\mathrm{D}} = 0 \end{aligned}$$

6 $$\begin{aligned}&g^{\mathrm{V}}_{u_{ii}} = g^{\mathrm{V}}_{d_{ii}} \equiv g_\mathrm{SM} \quad \mathrm{and}\quad g^{\mathrm{A}}_{u_{ii}} = g^{\mathrm{A}}_{d_{ii}} = 0 \end{aligned}$$

and

7 $$\begin{aligned}&g^\mathrm{V}_{X_\mathrm{D}} = 0 \quad \mathrm{and}\quad g^\mathrm{A}_{X_\mathrm{D}} \equiv g_X\end{aligned}$$

8 $$\begin{aligned}&g^{\mathrm{V}}_{u_{ii}} = g^{\mathrm{V}}_{d_{ii}} = 0 \quad \mathrm{and}\quad g^{\mathrm{A}}_{u_{ii}} = g^{\mathrm{A}}_{d_{ii}} \equiv g_\mathrm{SM}, \end{aligned}$$

respectively, where we assume quark couplings to the mediator to be flavour universal and set all flavour off-diagonal couplings to zero. With this simplification of a single universal coupling for the SM-$Y_1$ interactions, the model has only four independent parameters, i.e. two couplings and two masses:

9 $$\begin{aligned} \{g_\mathrm{SM},\,g_X,\,m_X,\,m_Y\}. \end{aligned}$$

We note that the mediator width is calculated from the above parameters.

Finding a signal of DM in this parameter space (or to constrain these parameters) is the primary goal of the DM searches at the LHC Run II [5], and the most important signature in this model is mono-jet plus MET. The di-jet final state via the $Y_1$ Drell–Yan process can be an important complementary channel.

### Spin-0 mediator model

Similarly, in the case of a spin-0 mediator ($Y_0$) interacting with the Dirac fermion DM and the SM particles, we define the interaction part of the Lagrangians as

10 $$\begin{aligned} \mathcal{L}_{X_\mathrm{D}}^{Y_0} = \bar{X}_\mathrm{D} (g^{\mathrm{S}}_{X_\mathrm{D}}+ig^{\mathrm{P}}_{X_\mathrm{D}}\gamma _5)X_\mathrm{D}\, Y_0, \end{aligned}$$

and

11 $$\begin{aligned} \mathcal{L}_\mathrm{SM}^{Y_0}&= \sum _{i,j} \Bigg [\bar{d}_i \frac{y_{ij}^d}{\sqrt{2}} (g^{\mathrm{S}}_{d_{ij}}+ig^{\mathrm{P}}_{d_{ij}}\gamma _5)d_j \nonumber \\&\quad + \bar{u}_i \frac{y_{ij}^u}{\sqrt{2}} (g^{\mathrm{S}}_{u_{ij}}+ig^{\mathrm{P}}_{u_{ij}}\gamma _5)u_j\Bigg ] Y_0, \end{aligned}$$

where $g^{\mathrm{S/P}}$ are the scalar/pseudo-scalar couplings of DM and quarks. Assuming a UV complete description of the scalar theory with the couplings of the mediator to the SM particles proportional to the particle masses, we normalise these couplings to the SM Yukawa couplings, $y_{ii}^f = \sqrt{2}m_f/v$, and set all flavour off-diagonal couplings to zero. This implies that, in a five-flavour scheme with massless bottom quarks, only top quarks are relevant for DM production in this model. Extension to a four-flavour scheme with massive bottom quarks is possible. The model file for the spin-0 mediator case, including other choices for the spin of the DM particle (real scalar $X_\mathrm{R}$ and complex scalar $X_\mathrm{C}$), is also available at the FeynRules repository [28].

The pure scalar and pure pseudo-scalar mediator scenarios are given by setting the parameters in the Lagrangians (10) and (11) to

12 $$\begin{aligned}&g^\mathrm{S}_{X_\mathrm{D}} \equiv g_X \quad \mathrm{and}\quad g^{\mathrm {P}}_{X_\mathrm{D}} = 0 \end{aligned}$$

13 $$\begin{aligned}&g^{\mathrm{S}}_{u_{33}} \equiv g_\mathrm{SM} \quad \mathrm{and}\quad g^{\mathrm{P}}_{u_{33}} = 0 \end{aligned}$$

and

14 $$\begin{aligned}&g^\mathrm{S}_{X_\mathrm{D}} = 0 \quad \mathrm{and}\quad g^\mathrm{P}_{X_\mathrm{D}} \equiv g_X\end{aligned}$$

15 $$\begin{aligned}&g^{\mathrm{S}}_{u_{33}} = 0 \quad \mathrm{and}\quad g^{\mathrm{P}}_{u_{33}} \equiv g_\mathrm{SM}, \end{aligned}$$

respectively. All the other $g^{\mathrm{S/P}}_{u_{ij}}$ and $g^{\mathrm{S/P}}_{d_{ij}}$ parameters are irrelevant. Similar to the spin-1 case, the model has only four independent parameters as in (9).

In the spin-0 mediator model with Yukawa-type couplings, the most relevant tree-level process at the LHC is DM pair production associated with a top-quark pair. On the other hand similarly to Higgs production, at one loop, gluon fusion can give rise to MET + jets signatures which are in general phenomenologically relevant. For the heavy mediator case, the four-top final state can be also relevant.

At this stage, we do not see compelling reasons to introduce couplings of the mediator to leptons, even though it is straightforward to do. We do not include effective gluon–gluon–$Y_0$ interactions either, for several reasons. The first is that this operator is higher dimensional ($\mathrm{dim} = 5$) and therefore might lead to unitarity-violating effects that need to be studied on a model and benchmark basis. The second is that a simplified model assumes no other new physics particle beyond the dark matter particle *X* and mediator *Y* at the weak scale. If such new particle effects decouple with their mass, the main contribution to the gluon–gluon–$Y_0$ coupling would then be due to loop of SM quarks. Depending on the masses involved (that of the DM, the mediator and the quarks contributing in the loop) and the momentum transfer of the process, such interactions might be considered point-like. This is, however, very much model and process dependent. To be accurate one should first calculate the loop-induced processes exactly and study the range of applicability of such an effective interaction by explicit comparison. This is possible in MG5aMC [43] and has been considered with the same DM implementation as presented here in Ref. [44]. Other studies of the loop-induced process for mono-jet + MET can be found in Refs. [45–48]. We also note that couplings of the mediator to the SM gauge bosons can be introduced easily [42].

## Dark matter production with jets

In this section, we consider a spin-1 mediator scenario and discuss the impact of the NLO-QCD corrections on DM pair production with jets, i.e.,

16 $$\begin{aligned} pp \rightarrow X \bar{X} + j(j). \end{aligned}$$

In MG5aMC the code and events for the above process can be automatically generated by issuing the following commands: 
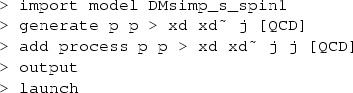


We have checked that our model can reproduce the SM predictions for $pp\rightarrow Zj(j)\rightarrow \tau ^+\tau ^-j(j)$ by adjusting the corresponding coupling and mass parameters.

To illustrate the effect of the higher-order corrections, we consider pure vector, Eqs. (5) and (6), or pure axial-vector, Eqs. (7) and (8), couplings with a simplified flavour structure. We take

17 $$\begin{aligned} g_X=1 \quad \mathrm{and}\quad g_\mathrm{SM}=0.25 \end{aligned}$$

as our benchmark for the spin-1 mediator scenario.

We assume that the mediator can only decay into the DM particle and the SM quarks (if kinematically allowed) through the interactions specified in Eqs. (3) and (4), so that the mediator width, $\Gamma _{Y}$, is determined by the particle masses and the couplings $g_{X}$ and $g_\mathrm{SM}$. In our framework, the width is automatically computed by using the MadWidth module [49] for each parameter point. The above benchmark coupling strength in (17) leads to $\Gamma _Y/m_Y\sim 0.05$ for $m_Y>2m_X$ and $\Gamma _Y/m_Y\sim 0.025$ for $m_Y<2m_X$, both for the vector and axial-vector cases. Note that, if we take $g_\mathrm{SM}=1 \ (0.5)$ with $g_X=1$, the $Y_1$ width becomes very large as $\Gamma _Y/m_Y\sim 0.5\ (0.15)$ for $m_Y>2m_X$.

We provide LO and NLO QCD predictions for $pp\rightarrow X\bar{X}+j(j)$ at the center-of-mass energy $\sqrt{s}=13$ TeV. The central value $\mu _0$ for the renormalisation ($\mu _\mathrm{R}$) and factorisation ($\mu _\mathrm{F}$) scales is set to $H_\mathrm{T}/2$, where $H_\mathrm{T}$ is the sum of the transverse momenta of all jets in the event and the missing transverse energy. The scale uncertainty is estimated by varying the scales $\mu _\mathrm{R}$ and $\mu _\mathrm{F}$, independently, by a factor two around $\mu _0$. We adopt the five-flavour scheme and the LO and NLO NNPDF2.3 set [50] through the LHAPDF interface [51], with the corresponding values of $\alpha _{s}^\mathrm{LO}(M_Z) = 0.130$ and $\alpha _{s}^\mathrm{NLO}(M_Z) = 0.118$, for the LO and NLO predictions, respectively. The PDF uncertainties are computed automatically [52], following the prescription summarised in [53].

Where relevant, we apply a parton shower to the events using Pythia8 [35]. We then define jets using the anti-$k_\mathrm{T}$ algorithm [54] as implemented in FastJet [55] with the jet cone radius $R=0.4$, where we require $p_{T}(j)>30$ GeV and $|\eta (j)|<4.5$ for all jets in the event.

### Total cross sections

**Fig. 1:**
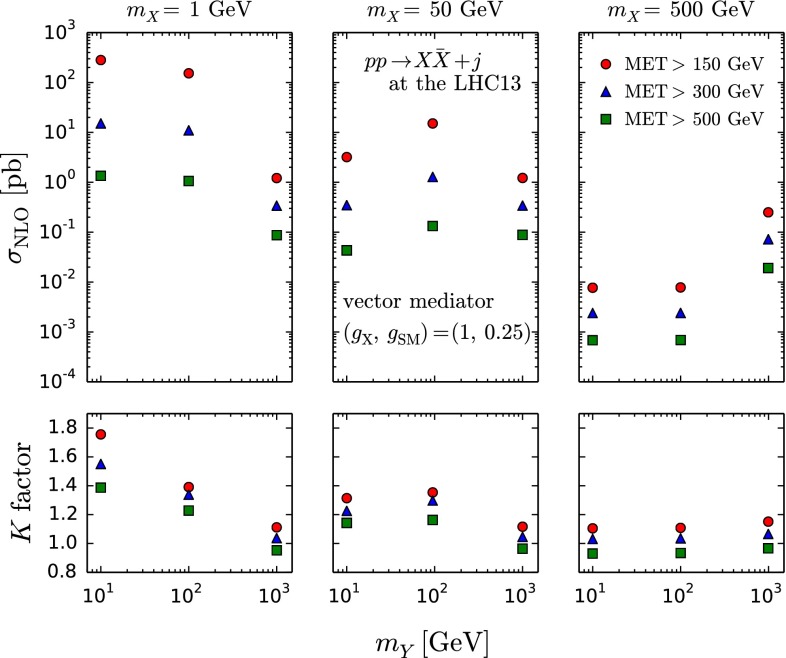
Summary plot of NLO cross sections and corresponding *K* factors in Table 1

In Table 1 we present LO and NLO cross sections (in pb) for DM pair production in association with a jet at fixed order (FO) in perturbation theory. We show results for a pure vector mediator by fixing the parameters as in Eqs. (5), (6) and (17). We cover various benchmark points suggested by the ATLAS/CMS DM forum [5] in the $m_{Y}$–$m_{X}$ plane, representing four different cases: on-shell ($m_{Y}>2m_{X}$) and off-shell ($m_{Y}<2m_{X}$) production of the mediator, in the threshold regime ($m_{Y}\lesssim 2m_{X}$) and in the EFT limit ($m_{Y}\gg \sqrt{\hat{s}}$). We also present scale and PDF uncertainties in % as well as *K* factors which we define as the ratio of the central values of the NLO and LO cross sections. We compute the table entries with different MET cuts: 150, 300, and 500 GeV. For convenience, we also show a graphical summary of our results in Fig. 1. As a reference, the cross sections for $pp\rightarrow Y_1+j$ are also shown, where the vector mediator $Y_1$ is produced on-shell and does not decay. For $m_{Y}>2m_{X}$, the mono-jet rate is given by $\sigma (pp\rightarrow X\bar{X}+j)\sim \sigma (pp\rightarrow Y_1+j)\times B(Y_1\rightarrow X\bar{X})$ in the narrow width approximation.

**Table 1:** LO and NLO cross sections and corresponding *K* factors for DM pair production in association with a jet for the vector mediator scenario at the 13-TeV LHC, where different MET cuts are imposed. The uncertainties represent the scale and PDF uncertainties in per cent, respectively. We show several benchmark model points for the mediator and DM masses with the coupling parameters $g_{X}=1$ and $g_\mathrm{SM}=0.25$

$(m_{Y},m_{X})$ [GeV]			Vector		
			MET $>150$ GeV	MET $>300$ GeV	MET $>500$ GeV
		$\sigma _{\mathrm{LO}}$ [pb]	${2.923\times 10^{2}\,}^{+10.7}_{-8.9}$ ${\pm 1.6\,\%}$	${1.734\times 10^{1}\,}^{+14.2}_{-11.9}$ ${\pm 1.1\,\%}$	${1.695\times 10^{0}\,}^{+17.4}_{-14.0}$ ${\pm 1.8\,\%}$
10	Undecayed	$\sigma _{\mathrm{NLO}}$ [pb]	${5.093\times 10^{2}\,}^{+10.3}_{-8.2}$ ${\pm 0.5\,\%}$	${2.689\times 10^{1}\,}^{+10.4}_{-9.1}$ ${\pm 0.6\,\%}$	${2.433\times 10^{0}\,}^{+11.1}_{-10.0}$ ${\pm 1.1\,\%}$
		*K* factor	1.74	1.55	1.44
		$\sigma _{\mathrm{LO}}$ [pb]	${1.605\times 10^{2}\,}^{+10.7}_{-8.9}$ ${\pm 1.6\,\%}$	${0.978\times 10^{1}\,}^{+14.3}_{-12.0}$ ${\pm 1.1\,\%}$	${0.970\times 10^{0}\,}^{+17.4}_{-14.1}$ ${\pm 2.0\,\%}$
(10, 1)	$m_{Y}\!>\!2m_{X}$	$\sigma _{\mathrm{NLO}}$ [pb]	${2.818\times 10^{2}\,}^{+10.1}_{-8.1}$ ${\pm 0.5\,\%}$	${1.517\times 10^{1}\,}^{+10.0}_{-8.9}$ ${\pm 0.6\,\%}$	${1.345\times 10^{0}\,}^{+10.5}_{-9.6}$ ${\pm 1.1\,\%}$
		*K* factor	1.76	1.55	1.39
		$\sigma _{\mathrm{LO}}$ [pb]	${2.434\times 10^{0}\,}^{+11.8}_{-10.1}$ ${\pm 1.5\,\%}$	${2.843\times 10^{-1}\,}^{+15.0}_{-12.5}$ ${\pm 1.2\,\%}$	${3.786\times 10^{-2}\,}^{+18.0}_{-14.5}$ ${\pm 2.4\,\%}$
(10, 50)	$m_{Y}\!<\!2m_{X}$	$\sigma _{\mathrm{NLO}}$ [pb]	${3.198\times 10^{0}\,}^{+5.6}_{-5.4}$ ${\pm 0.5\,\%}$	${3.485\times 10^{-1}\,}^{+5.9}_{-6.3}$ ${\pm 0.7\,\%}$	${4.325\times 10^{-2}\,}^{+7.3}_{-7.8}$ ${\pm 1.3\,\%}$
		*K* factor	1.31	1.23	1.14
		$\sigma _{\mathrm{LO}}$ [pb]	${6.968\times 10^{-3}\,}^{+17.4}_{-14.0}$ ${\pm 4.3\,\%}$	${2.314\times 10^{-3}\,}^{+18.9}_{-15.0}$ ${\pm 4.6\,\%}$	${7.317\times 10^{-4}\,}^{+20.6}_{-16.1}$ ${\pm 5.6\,\%}$
(10, 500)	$m_{Y}\!<\!2m_{X}$	$\sigma _{\mathrm{NLO}}$ [pb]	${7.698\times 10^{-3}\,}^{+5.4}_{-6.4}$ ${\pm 2.2\,\%}$	${2.385\times 10^{-3}\,}^{+5.7}_{-6.9}$ ${\pm 2.3\,\%}$	${6.800\times 10^{-4}\,}^{+5.5}_{-7.1}$ ${\pm 2.6\,\%}$
		*K* factor	1.10	1.03	0.93
		$\sigma _{\mathrm{LO}}$ [pb]	${2.148\times 10^{2}\,}^{+10.6}_{-9.3}$ ${\pm 1.5\,\%}$	${1.616\times 10^{1}\,}^{+14.4}_{-12.0}$ ${\pm 1.0\,\%}$	${1.644\times 10^{0}\,}^{+17.4}_{-14.1}$ ${\pm 1.9\,\%}$
100	Undecayed	$\sigma _{\mathrm{NLO}}$ [pb]	${3.011\times 10^{2}\,}^{+6.6}_{-5.9}$ ${\pm 0.5\,\%}$	${2.121\times 10^{1}\,}^{+7.3}_{-7.1}$ ${\pm 0.6\,\%}$	${1.955\times 10^{0}\,}^{+8.1}_{-8.2}$ ${\pm 1.2\,\%}$
		*K* factor	1.40	1.31	1.19
		$\sigma _{\mathrm{LO}}$ [pb]	${1.100\times 10^{2}\,}^{+10.6}_{-9.3}$ ${\pm 1.5\,\%}$	${0.822\times 10^{1}\,}^{+14.4}_{-12.0}$ ${\pm 1.1\,\%}$	${0.862\times 10^{0}\,}^{+17.4}_{-14.1}$ ${\pm 1.9\,\%}$
(100, 1)	$m_{Y}\!>\!2m_{X}$	$\sigma _{\mathrm{NLO}}$ [pb]	${1.530\times 10^{2}\,}^{+6.5}_{-5.7}$ ${\pm 0.5\,\%}$	${1.100\times 10^{1}\,}^{+7.4}_{-7.2}$ ${\pm 0.6\,\%}$	${1.059\times 10^{0}\,}^{+8.0}_{-8.1}$ ${\pm 1.2\,\%}$
		*K* factor	1.39	1.34	1.23
		$\sigma _{\mathrm{LO}}$ [pb]	${1.117\times 10^{1}\,}^{+11.0}_{-9.6}$ ${\pm 1.5\,\%}$	${0.988\times 10^{0}\,}^{+14.7}_{-12.2}$ ${\pm 1.1\,\%}$	${1.140\times 10^{-1}\,}^{+17.6}_{-14.2}$ ${\pm 2.0\,\%}$
(95, 50)	$m_{Y}\!\lesssim \!2m_{X}$	$\sigma _{\mathrm{NLO}}$ [pb]	${1.512\times 10^{1}\,}^{+6.0}_{-5.5}$ ${\pm 0.5\,\%}$	${1.281\times 10^{0}\,}^{+6.8}_{-6.8}$ ${\pm 0.6\,\%}$	${1.325\times 10^{-1}\,}^{+7.2}_{-7.6}$ ${\pm 1.2\,\%}$
		*K* factor	1.35	1.30	1.16
		$\sigma _{\mathrm{LO}}$ [pb]	${7.043\times 10^{-3}\,}^{+17.4}_{-14.0}$ ${\pm 4.3\,\%}$	${2.329\times 10^{-3}\,}^{+18.9}_{-15.0}$ ${\pm 4.6\,\%}$	${7.395\times 10^{-4}\,}^{+20.6}_{-16.1}$ ${\pm 5.6\,\%}$
(100, 500)	$m_{Y}\!<\!2m_{X}$	$\sigma _{\mathrm{NLO}}$ [pb]	${7.804\times 10^{-3}\,}^{+5.3}_{-6.4}$ ${\pm 2.2\,\%}$	${2.411\times 10^{-3}\,}^{+5.5}_{-6.8}$ ${\pm 2.3\,\%}$	${6.908\times 10^{-4}\,}^{+5.5}_{-7.1}$ ${\pm 2.6\,\%}$
		*K* factor	1.11	1.04	0.93
		$\sigma _{\mathrm{LO}}$ [pb]	${2.248\times 10^{0}\,}^{+16.1}_{-13.2}$ ${\pm 3.2\,\%}$	${6.865\times 10^{-1}\,}^{+17.7}_{-14.3}$ ${\pm 3.3\,\%}$	${1.979\times 10^{-1}\,}^{+19.6}_{-15.5}$ ${\pm 4.1\,\%}$
1000	Undecayed	$\sigma _{\mathrm{NLO}}$ [pb]	${2.601\times 10^{0}\,}^{+5.1}_{-6.0}$ ${\pm 1.7\,\%}$	${7.393\times 10^{-1}\,}^{+5.2}_{-6.4}$ ${\pm 1.8\,\%}$	${1.909\times 10^{-1}\,}^{+5.3}_{-6.8}$ ${\pm 2.1\,\%}$
		*K* factor	1.16	1.08	0.96
		$\sigma _{\mathrm{LO}}$ [pb]	${1.093\times 10^{0}\,}^{+16.4}_{-13.3}$ ${\pm 3.1\,\%}$	${3.278\times 10^{-1}\,}^{+18.0}_{-14.4}$ ${\pm 3.3\,\%}$	${9.182\times 10^{-2}\,}^{+19.7}_{-15.6}$ ${\pm 4.1\,\%}$
(1000, 1)	$m_{Y}\!>\!2m_{X}$	$\sigma _{\mathrm{NLO}}$ [pb]	${1.215\times 10^{0}\,}^{+4.2}_{-5.5}$ ${\pm 1.7\,\%}$	${3.399\times 10^{-1}\,}^{+4.5}_{-6.0}$ ${\pm 1.7\,\%}$	${8.743\times 10^{-2}\,}^{+4.8}_{-6.5}$ ${\pm 2.0\,\%}$
		*K* factor	1.11	1.04	0.95
		$\sigma _{\mathrm{LO}}$ [pb]	${1.094\times 10^{0}\,}^{+16.4}_{-13.3}$ ${\pm 3.1\,\%}$	${3.268\times 10^{-1}\,}^{+18.0}_{-14.4}$ ${\pm 3.3\,\%}$	${9.137\times 10^{-2}\,}^{+19.7}_{-15.6}$ ${\pm 4.1\,\%}$
(1000, 50)	$m_{Y}\!>\!2m_{X}$	$\sigma _{\mathrm{NLO}}$ [pb]	${1.221\times 10^{0}\,}^{+4.3}_{-5.6}$ ${\pm 1.7\,\%}$	${3.416\times 10^{-1}\,}^{+4.6}_{-6.0}$ ${\pm 1.7\,\%}$	${8.807\times 10^{-2}\,}^{+4.9}_{-6.6}$ ${\pm 2.0\,\%}$
		*K* factor	1.12	1.05	0.96
		$\sigma _{\mathrm{LO}}$ [pb]	${2.169\times 10^{-1}\,}^{+16.4}_{-13.3}$ ${\pm 3.4\,\%}$	${6.777\times 10^{-2}\,}^{+18.0}_{-14.4}$ ${\pm 3.6\,\%}$	${1.981\times 10^{-2}\,}^{+19.7}_{-15.6}$ ${\pm 4.4\,\%}$
(995, 500)	$m_{Y}\!\lesssim \!2m_{X}$	$\sigma _{\mathrm{NLO}}$ [pb]	${2.497\times 10^{-1}\,}^{+5.3}_{-6.2}$ ${\pm 1.8\,\%}$	${7.223\times 10^{-2}\,}^{+5.5}_{-6.6}$ ${\pm 1.9\,\%}$	${1.914\times 10^{-2}\,}^{+5.3}_{-6.8}$ ${\pm 2.1\,\%}$
		*K* factor	1.15	1.07	0.97
		$\sigma _{\mathrm{LO}}$ [pb]	${8.487\times 10^{-6}\,}^{+18.0}_{-14.3}$ ${\pm 4.3\,\%}$	${2.666\times 10^{-6}\,}^{+20.0}_{-15.7}$ ${\pm 5.5\,\%}$	${8.238\times 10^{-7}\,}^{+22.0}_{-17.0}$ ${\pm 7.3\,\%}$
(10000, 1)	$m_{Y}\!\gg \!\sqrt{\hat{s}}$	$\sigma _{\mathrm{NLO}}$ [pb]	${8.835\times 10^{-6}\,}^{+3.1}_{-5.1}$ ${\pm 2.5\,\%}$	${2.579\times 10^{-6}\,}^{+3.1}_{-5.5}$ ${\pm 3\,\%}$	${7.148\times 10^{-7}\,}^{+5.0}_{-7.0}$ ${\pm 4.4\,\%}$
		*K* factor	1.04	0.97	0.87

The production rate strongly depends on the both masses as well as on the kinematic cuts, and varies by orders of magnitude in the parameter scan. On the other hand, the *K* factors, i.e. higher-order effects, are not so sensitive to the mass spectra; $K\sim 1.1$ for the heavy-mediator and/or heavy-DM cases, while $K\sim 1.3$–1.4 for the ${\sim } 100 \mathrm{\ GeV}$ mediator with light DM, assuming the $\mathrm{MET}>150$ GeV cut. We find that in the case of a relatively light mediator with a very light DM, $(m_Y,m_X)=(10,1)$ GeV, the *K* factor can reach a value as large as 1.8.

Different benchmark points probe different Bjorken-*x* regions of the parton distribution functions. As heavy mediators/DM are produced from very high-*x* partons, the dominant contribution comes from the $q\bar{q}$ initial state, as the gluon PDF is sub-dominant in the high-*x* region. For light mediators with light DM, on the other hand, a large contribution arises from the *qg* initial state. For instance, we find that the ratio of production cross sections via $u\bar{u}$ and *ug* initial states, $\sigma (u\bar{u}) / \sigma (ug)$, is 1.4 in the case of $(m_Y, m_X)=(1000, 50) \mathrm{\ GeV}$ while 0.2 for $(m_Y, m_X)=(100, 1) \mathrm{\ GeV}$, at LO.

As expected, most of the results at NLO accuracy display significantly smaller scale uncertainties compared to the LO calculations. An exception is provided by the $(m_Y,m_X)=(10,1)$ GeV case, which we discuss in detail at the end of this subsection. The PDF uncertainties are sub-leading in both the LO and NLO results and reduced by going from LO to NLO. Furthermore, the scale and PDF uncertainties increase when the mass scale of the mediator and/or DM increases.

**Fig. 2:**
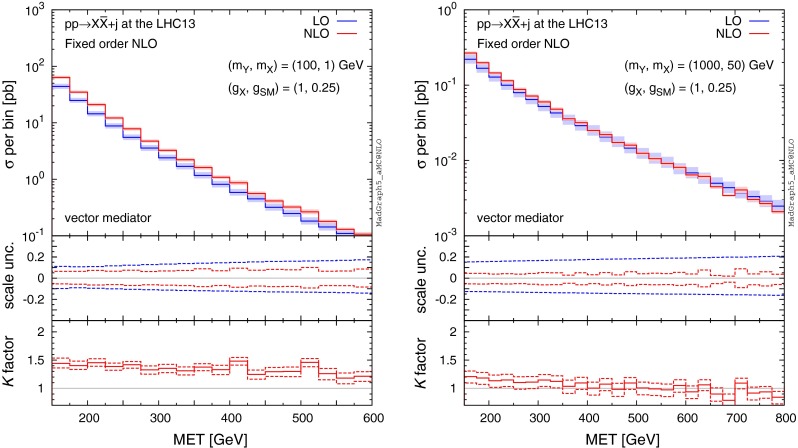
MET distributions at FO (N)LO accuracy for $pp\rightarrow X\bar{X}+j$ at the 13-TeV LHC for $(m_Y,m_X)=(100,1)$ and (1000, 50) GeV, where we assume a pure vector mediator and Dirac DM. The *middle and bottom panels* show the differential scale uncertainties and *K* factors, respectively

The higher $\mathrm{MET}$ cut leads to smaller *K* factors and to larger scale and PDF uncertainties, which one can clearly see in the MET distributions in Fig. 2.

In Table 2, we present the pure axial-vector mediator case by fixing the parameters as in Eqs. (7), (8) and (17). The resulting cross sections are very similar compared to the pure vector case for $m_Y>2m_X$, while in the off-shell regime, we find that the cross sections are suppressed compared to the production via pure vector mediators. In the off-shell situation the DM pair is produced at threshold. A pair of (Dirac) DM originating from a decay of a spin-1 mediator will be in a $~^{2S+1}L_J$ state with $J=1$. If the coupling is vector-like the DM pair can be in a $^3S_1$ state, while if it is axial-like it will be in a $^3P_1$ state, i.e. suppressed at threshold. The similar argument holds in case of $gg\rightarrow Y_0+ t\bar{t}$ case, as we show in Sect. 4.

**Table 2:** Same as Table 1, but for the axial-vector mediator scenario

$(m_{Y},m_{X})$ [GeV]			Axial-vector		
			MET $>150$ GeV	MET $>300$ GeV	MET $>500$ GeV
		$\sigma _{\mathrm{LO}}$ [pb]	${2.130\times 10^{2}\,}^{+10.6}_{-9.3}$ ${\pm 1.6\,\%}$	${1.573\times 10^{1}\,}^{+14.4}_{-12.0}$ ${\pm 1.1\,\%}$	${1.633\times 10^{0}\,}^{+17.3}_{-14.0}$ ${\pm 1.9\,\%}$
100	Undecayed	$\sigma _{\mathrm{NLO}}$ [pb]	${3.063\times 10^{2}\,}^{+6.9}_{-6.1}$ ${\pm 0.5\,\%}$	${2.153\times 10^{1}\,}^{+7.7}_{-7.4}$ ${\pm 0.6\,\%}$	${2.055\times 10^{0}\,}^{+8.4}_{-8.3}$ ${\pm 1.6\,\%}$
		*K* factor	1.44	1.37	1.26
		$\sigma _{\mathrm{LO}}$ [pb]	${1.101\times 10^{2}\,}^{+10.6}_{-9.3}$ ${\pm 1.6\,\%}$	${0.825\times 10^{1}\,}^{+14.4}_{-12.1}$ ${\pm 1.1\,\%}$	${0.854\times 10^{0}\,}^{+17.4}_{-14.1}$ ${\pm 2\,\%}$
(100, 1)	$m_{Y}\!>\!2m_{X}$	$\sigma _{\mathrm{NLO}}$ [pb]	${1.549\times 10^{2}\,}^{+6.8}_{-6.0}$ ${\pm 0.5\,\%}$	${1.127\times 10^{1}\,}^{+7.4}_{-7.2}$ ${\pm 0.6\,\%}$	${1.063\times 10^{0}\,}^{+8.2}_{-8.2}$ ${\pm 1.2\,\%}$
		*K* factor	1.41	1.37	1.24
		$\sigma _{\mathrm{LO}}$ [pb]	${3.070\times 10^{0}\,}^{+11.6}_{-10.0}$ ${\pm 1.5\,\%}$	${3.359\times 10^{-1}\,}^{+14.9}_{-12.4}$ ${\pm 1.2\,\%}$	${4.457\times 10^{-2}\,}^{+17.7}_{-14.3}$ ${\pm 1.8\,\%}$
(95, 50)	$m_{Y}\!\lesssim \!2m_{X}$	$\sigma _{\mathrm{NLO}}$ [pb]	${4.093\times 10^{0}\,}^{+6.0}_{-5.7}$ ${\pm 0.5\,\%}$	${4.302\times 10^{-1}\,}^{+6.7}_{-6.9}$ ${\pm 0.7\,\%}$	${5.079\times 10^{-2}\,}^{+6.9}_{-7.4}$ ${\pm 1.3\,\%}$
		*K* factor	1.33	1.28	1.14
		$\sigma _{\mathrm{LO}}$ [pb]	${2.298\times 10^{-3}\,}^{+18.1}_{-14.5}$ ${\pm 5\,\%}$	${7.839\times 10^{-4}\,}^{+19.5}_{-15.4}$ ${\pm 5.3\,\%}$	${2.558\times 10^{-4}\,}^{+21.2}_{-16.5}$ ${\pm 6.3\,\%}$
(100, 500)	$m_{Y}\!<\!2m_{X}$	$\sigma _{\mathrm{NLO}}$ [pb]	${2.502\times 10^{-3}\,}^{+5.9}_{-6.8}$ ${\pm 2.5\,\%}$	${7.972\times 10^{-4}\,}^{+6.2}_{-7.3}$ ${\pm 2.6\,\%}$	${2.383\times 10^{-4}\,}^{+6.1}_{-7.5}$ ${\pm 3.0\,\%}$
		*K* factor	1.09	1.02	0.93

The NLO effects are very similar to the vector mediator scenario for all the mass combinations as well as the $\mathrm{MET}$ cuts. Although we do not show the mixed scenario of vector and axial-vector, one can easily compute such scenarios by changing the coupling parameters in our simplified model.

The parameter point $(m_Y,m_X)=(10,1)$ GeV warrants special attention, as it illustrates a case of large NLO corrections (so-called “giant *K* factors” [56]), which might arise in the limit where $p_\mathrm{T}^j \gg m_{Y},m_{X}$. The giant *K* factors in the $p p \rightarrow Y_1+j$ process occur due to the opening of the $pp\rightarrow Y_1+jj$ channel at NLO. This process can lead to a di-jet event topology with a soft, collinear emission of $Y_1$. In the regime of $p_\mathrm{T}^j \gg m_Y$, the $Y_1$ emission behaves similar to an emission of a massless gauge boson, where the diagrams with dijet topologies contribute factors of $\alpha _{s}^2 g_X^2\mathrm {log}^2(p_\mathrm{T}^j/m_Y)$, and hence NLO *K* factors which scale as $\sim \alpha _{s} \mathrm {log}^2(p_\mathrm{T}^j/m_Y)$. Similar features commonly appear in calculations of SM *W* / *Z*+jets processes at high jet $p_\mathrm{T}$ [56].

**Fig. 3:**
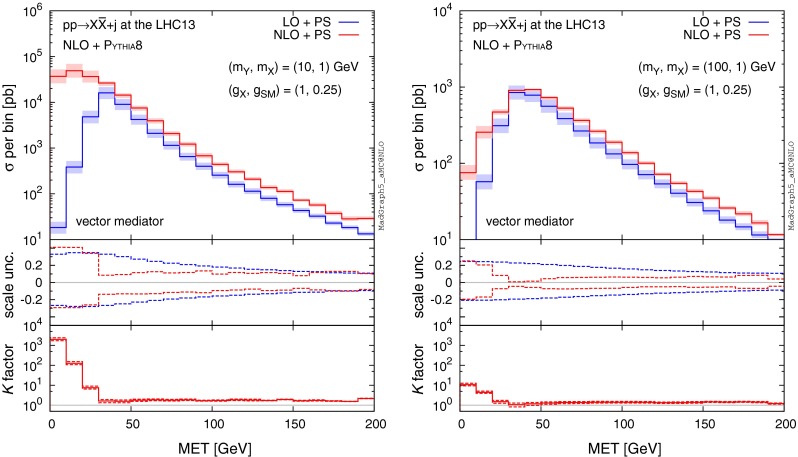
MET distribution at (N)LO+PS accuracy for $pp\rightarrow X\bar{X}+j$ at the 13-TeV LHC for $(m_Y,m_X)=(10,1) \mathrm{\ GeV}$ (*left*) and $(m_Y,m_X)=(100,1) \mathrm{\ GeV}$ (*right*). The *lower panels* provide information on the differential scale uncertainty and *K* factor

Topologies leading to giant *K* factors are naturally suppressed in the case of DM production by the cut on MET. This restricts the calculation to regions of phase space which are insensitive to the soft and collinear double-logs of di-jet event topologies with a soft $Y_1$ emission. Figure 3 (left) illustrates the effect in case of $m_Y=10$ GeV and $m_{X}=1$ GeV. The region of low missing energy displays a two to three orders of magnitude difference in rate between the LO and NLO calculations, whereas we see that above $\mathrm{MET}> 50 \mathrm{\ GeV}$, the *K* factor is drastically reduced. On the other hand, we see that already for $(m_Y,m_X)=(100,1)$ GeV in Fig. 3 (right), such logarithmic enhancements are only very weak.

### Differential distributions

**Fig. 4:**
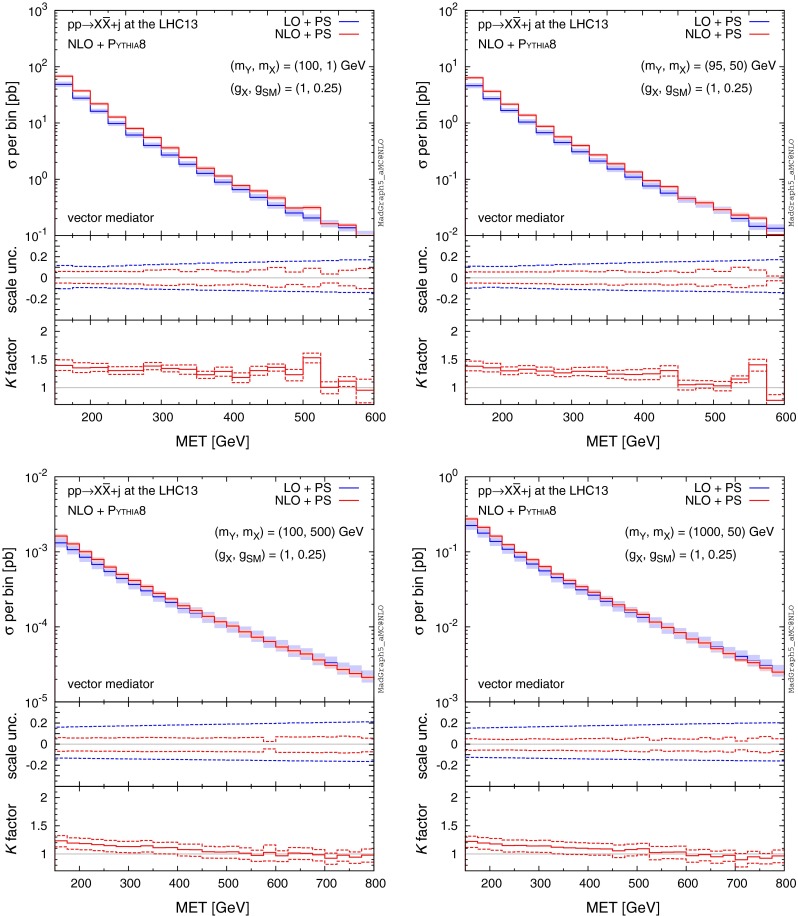
MET distributions for $pp\rightarrow X\bar{X}+j$ at the 13-TeV LHC for four benchmark points specified by ($m_Y,m_X$), where we assume a pure vector mediator and Dirac DM. The *middle and bottom panels* show the differential scale uncertainties and *K* factors, respectively

We proceed with the discussion of the features of the differential distributions relevant for DM studies. We begin with Fig. 4 which shows the $\mathrm{MET}$ distributions at LO and NLO for four benchmark points of the simplified model, assuming a pure vector mediator and Dirac fermion DM. As seen in the total rates, the NLO effects in the distributions do not depend on the mass relation between the mediator and the DM, i.e. on-shell or off-shell, but do depend on the energy scale of the final state. In the top panels, the energy scale is $\mathcal{O}(100)$ GeV for $m_Y$ or $2m_X$. We find that the two benchmark points display striking similarities in the shape of the $\mathrm{MET}$ distributions, while the rate of the latter is suppressed due to off-shell $Y_1$ production. The largest effects of NLO corrections are in the low $\mathrm{MET}$ regions, where NLO corrections reach *K* factors of about 1.4 for $\mathrm{MET}\sim 150 \mathrm{\ GeV}$, with a steady decrease with increasing $\mathrm{MET}$. We observe similar features also in the high-scale benchmark points of $\mathcal{O}(1)$ TeV for $m_Y$ or $2m_X$ (bottom panels of Fig. 4), where the largest *K* factors are about 1.2 for $\mathrm{MET}\sim 150 \mathrm{\ GeV}$. Comparing with the FO distributions in Fig. 2, we observe that the parton shower does not affect the MET distribution. Note that the NLO corrections are different for different $\mathrm{MET}$ regions, with the largest NLO corrections occurring in the lower MET regions where the rate is the highest. Hence the careful estimation of NLO effects is very important for accurate LHC studies of DM in each signal region.

**Fig. 5:**
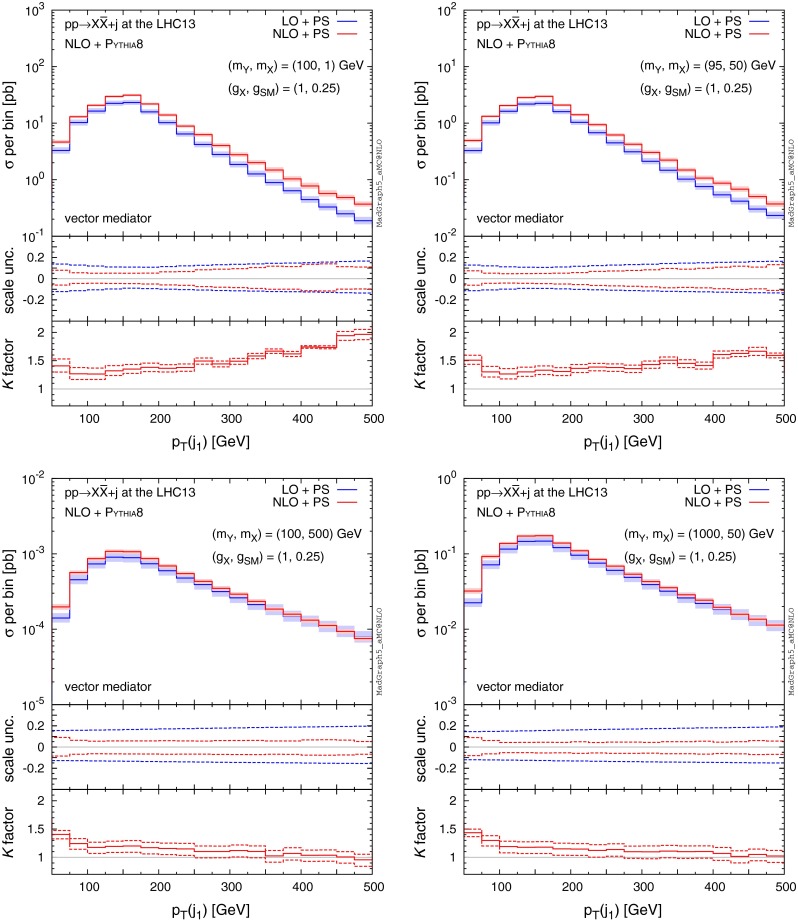
$p_\mathrm{T}$ distributions of the hardest jet for $pp\rightarrow X\bar{X}+j$ at the 13-TeV LHC for four benchmark points specified by ($m_Y,m_X$), where we assume a pure vector mediator and Dirac DM and the $\mathrm{MET}> 150 \mathrm{\ GeV}$ cut is imposed. The *middle and bottom panels* show the differential scale uncertainties and *K* factors, respectively

**Fig. 6:**
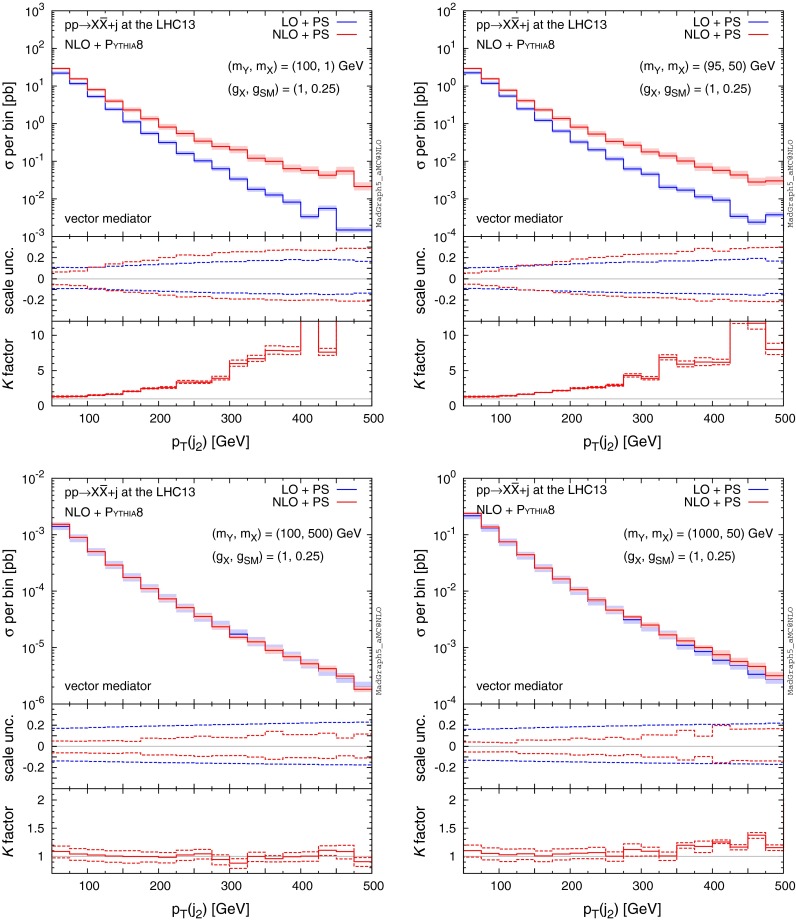
Same as Fig. 5, but for the second hardest jet

**Fig. 7:**
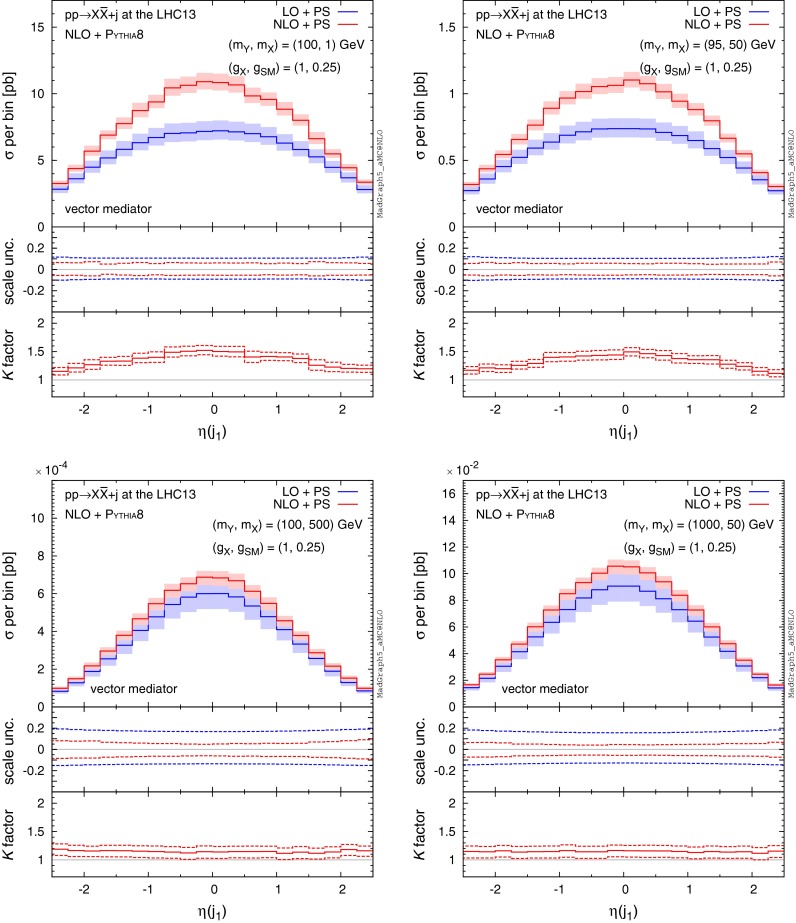
$\eta$ distributions of the hardest jet for $pp\rightarrow X\bar{X}+j$ at the 13-TeV LHC for four benchmark points specified by ($m_Y,m_X$), where we assume a pure vector mediator and Dirac DM and the $\mathrm{MET}> 150 \mathrm{\ GeV}$ cut is imposed. The *middle and bottom panels* show the differential scale uncertainties and *K* factors, respectively

**Fig. 8:**
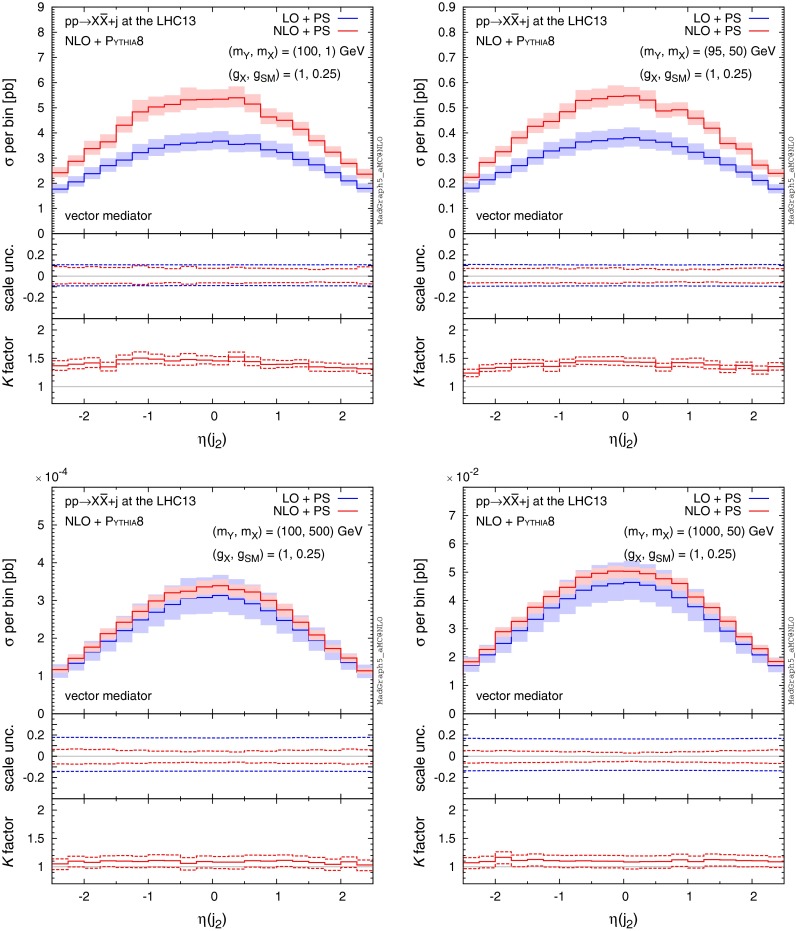
Same as Fig. 7, but for the second hardest jet

**Fig. 9:**
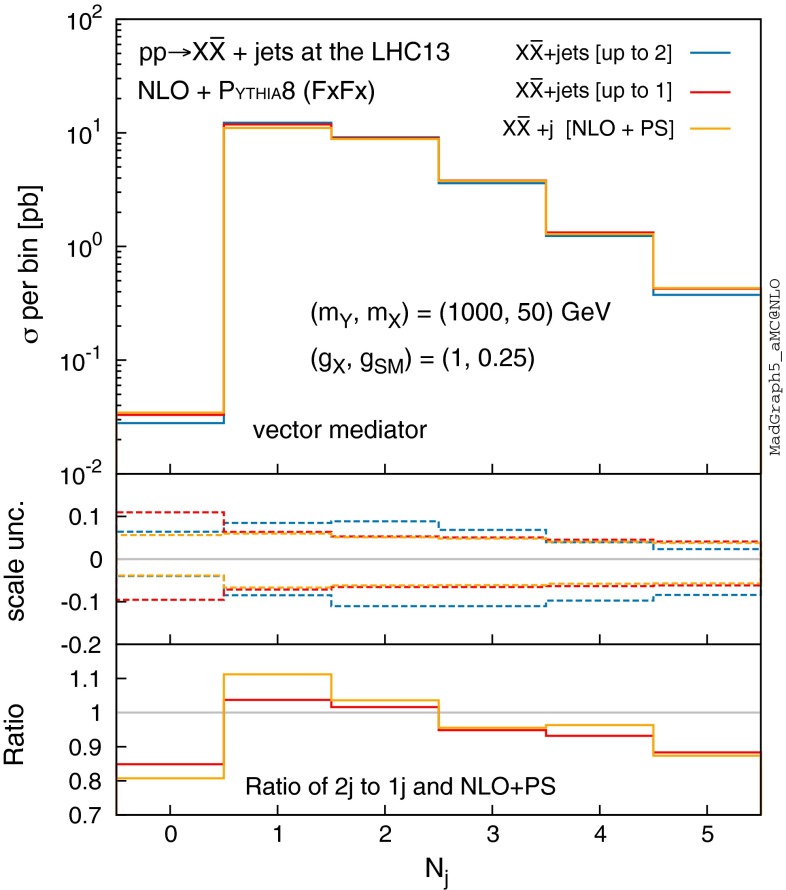
Jet multiplicity for $pp\rightarrow X\bar{X}+$ jets at the 13-TeV LHC with FxFx merging in case of $m_Y=1$ TeV with $m_X=50$ GeV, where NLO+PS samples are merged up to one (*red*) and two (*blue*) jets. The 1-jet NLO+PS sample without merging (*orange*) is also shown for comparison. The *middle panel* shows the relative scale uncertainties, while the *bottom panel* presents the ratio of the 2*j* merged sample to the 1*j* merged one and to the non-merged one. We assume $\mathrm{MET}>100 \mathrm{\ GeV}$ and jets with $p_\mathrm{T} > 30 \mathrm{\ GeV}$ and $|\eta | < 4.5$ for the purpose of illustration

Next, we study the features of jet kinematic distributions produced in association with DM. Figures 5, 6, 7 and 8 show example $p_\mathrm{T}$ and $\eta$ distributions of the hardest and second hardest jets for the four benchmark points as in Fig. 4, assuming

18 $$\begin{aligned} \mathrm{MET}>150\ \mathrm{GeV}. \end{aligned}$$

Distributions of the hardest jet transverse momentum show very interesting features. In Fig. 5 we find that, in all benchmark points, the LO distributions match the NLO predictions at the peak, i.e. $p_\mathrm{T}(j_1)\sim 150$ GeV, to a very good degree. The agreement can be attributed to the imposed $\mathrm{MET}$ cut in (18), which forces the events into a back-to-back configuration of the leading jet and the $Y_1$ mediator (on average). We also note that the NLO scale uncertainty in the peak region becomes very small compared to the LO estimates.

The NLO corrections to $p_\mathrm{T} (j_1)$ distributions affect not only the overall rate, but the shape of the distribution as well. In the low-$p_\mathrm{T}$ region, *K* factors are about 1.2–1.5. In the high $p_\mathrm{T}$ region, we find significant NLO effects again for the $(m_Y,m_X)=(100,1)$ and (95, 50) GeV cases (top panels), but not for the $(m_Y,m_X)=(100, 500)$ and (1000, 50) GeV cases (bottom panels). We note that the scale uncertainty does not significantly reduce at NLO in the $p_\mathrm{T}$ regions away from the peak, especially for the light mediator and DM case (top panels). Significant differences in NLO contributions and theoretical uncertainties in different regions of the $p_\mathrm{T}(j_1)$ spectrum suggest that the proper modelling of the hardest jet differential distributions has to go beyond the simple scaling by a constant *K* factor.

Apart from the highest $p_\mathrm{T}$ jet which is modelled by the hard matrix element, all other jets in the LO simulation are generated by the parton shower. By contrast, the NLO corrections include real emission diagrams which can contain two hard and well-separated partons in the final state as well as virtual corrections to one parton emission. One could expect significant differences between LO and NLO in the kinematic distributions of the second highest $p_\mathrm{T}$ jet. For the $(m_Y,m_X)=(100,1)$ and (95, 50) GeV cases (top panels), we observe giant *K* factors in the high-$p_\mathrm{T}$ tails of the distributions. The large difference between LO and NLO computations is a consequence of the inadequacy of the parton shower to accurately model high-$p_\mathrm{T}$ emissions. In Fig. 6 (bottom panels), on the other hand, we find no significant differences between LO and NLO for the overall rate and shape of the second jet emission in case of very heavy mediators (i.e. $m_Y = 1 \mathrm{\ TeV}$) or heavy DM (i.e. $m_X = 500 \mathrm{\ GeV}$), suggesting that the second hardest jet is described very well by the parton shower. This is because the scale of the shower is very high and therefore extra parton emission from the parton shower can be sufficiently hard.

Features similar to those observed in $p_\mathrm{T}(j_{1,2})$ also occur in distributions of the hardest/second-hardest jet pseudo-rapidity ($\eta (j_{1,2})$), shown in Figs. 7 and 8. For the light mediator/DM (top panels), we observe that the rate at which the hardest jet is emitted at NLO in the low rapidity region is enhanced by a factor about 1.5, with the corrections falling off with the increase in rapidity. However, even though the overall rate for the second-hardest jet increases by a factor of roughly ${\sim }1.5$ the shape of the $\eta (j_2)$ distribution is affected only mildly. In the case of heavy mediator/DM, the hardest jet is emitted at a lower rapidity (on average) at a significantly higher rate compared to light mediators as illustrated by the width of the $\eta$ distributions in Fig. 7. As the hardest jet typically recoils against $\mathrm{MET}$, this explains why the $\mathrm{MET}$ spectrum falls off more quickly for lighter mediators than for the heavier ones.

### Merging samples at NLO accuracy

In addition to total and differential production cross sections for the $pp\rightarrow X\bar{X}+j$ process, we study NLO effects for different jet multiplicities in the final state. For this purpose we utilise the FxFx merging procedure [20] within the framework of MG5aMC, and consider

19 $$\begin{aligned} pp\rightarrow X\bar{X} +0,1,2\ \mathrm {jets}. \end{aligned}$$

We take the merging scales at 45 and 30 GeV for 2- and 1-jet merged samples, respectively.

Figure 9 shows the number of jets in the final state for NLO merged samples in case of $m_{Y} = 1$ TeV and $m_{X} = 50$ GeV. The red and blue curves show the results of merging up to 1 and 2 jets, respectively, while the orange curve shows the $pp\rightarrow X\bar{X}+1j$ process at NLO+PS without merging for comparison. An inspection of the three samples in the lowest panel of Fig. 9 shows that the effects of NLO merging are mild. The non-merged NLO sample over-estimates the production rate in the 0*j* and ${>}2j$ bins by 20 and 10 % respectively, and underestimates the rate in the 1*j*–2*j* bins by ${<}10\,\%$. The differences are even milder between the samples merged to 1*j* and to 2*j*. As the 0*j* bin is phenomenologically irrelevant, we can conclude that the effects of jet merging at NLO are within $10\,\%$.

**Fig. 10:**
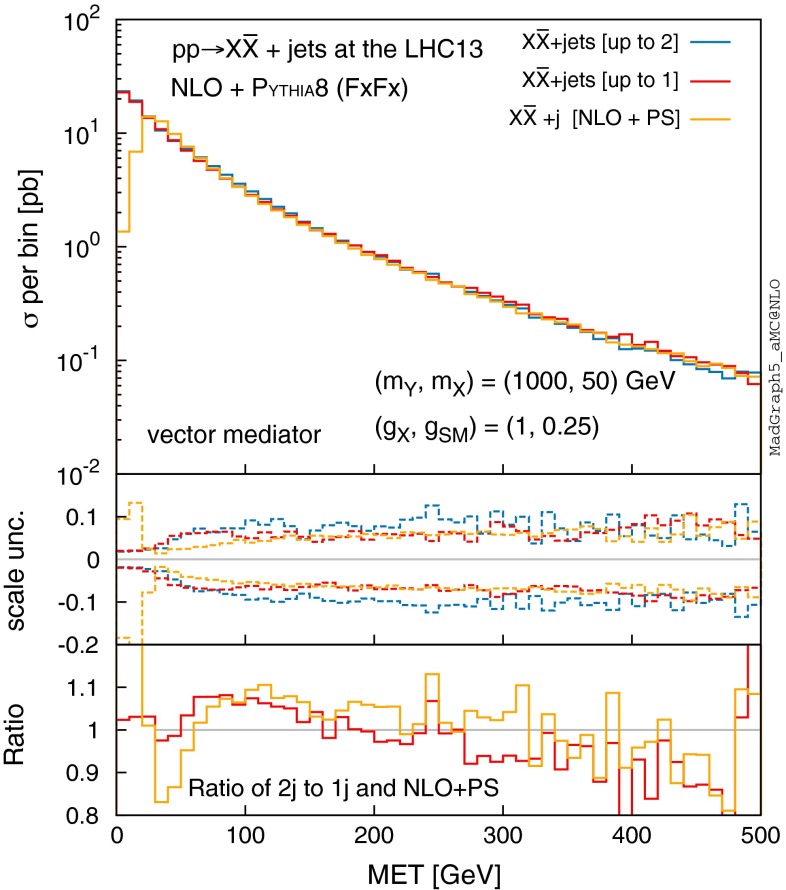
Same as Fig. 9, but for the MET distribution

We show effects of jet merging on the MET distribution in Fig. 10. Except in the low MET region, we find that the effects of NLO merging are again mild and within 10 %.

### Comparison of signal distributions to the Standard Model

In discussions of NLO corrections to DM production, it is important to consider how the possible signal events at NLO look in the midst of large SM backgrounds. For the purpose of illustration, we consider only the largest background in mono-jet searches for DM, i.e. *Z*+jets and simulate it to NLO merged up to 2 extra jets via the FxFx method.

**Fig. 11:**
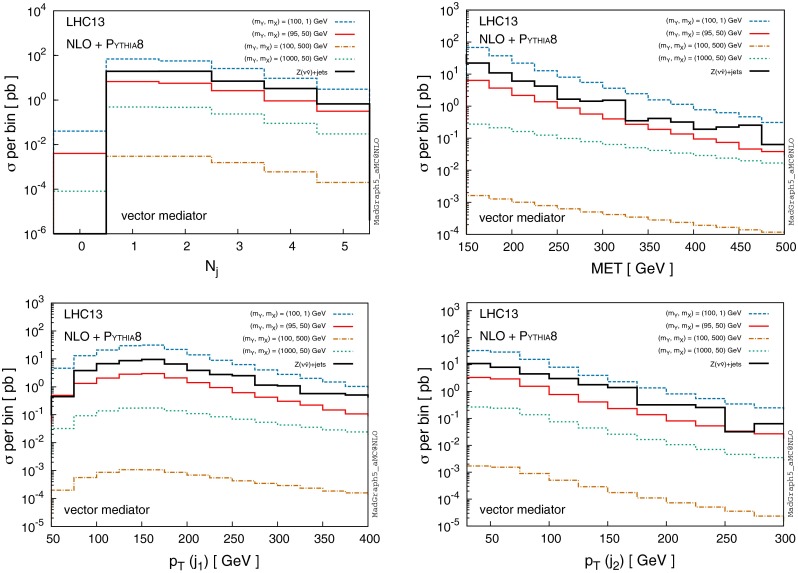
Distributions for various signal benchmark points and $Z(\nu \bar{\nu })+$ jets, where $\mathrm{MET}> 150 \mathrm{\ GeV}$ and jets with $p_\mathrm{T} > 30 \mathrm{\ GeV}$ and $|\eta | < 4.5$ are considered. We assume a pure vector mediator and Dirac DM with the coupling parameters $g_X=1$ and $g_\mathrm{SM}=0.25$

Figure 11 shows an example comparison of the *Z*+jets channel to several benchmark points of the simplified model discussed in previous sections. The shape of signal jet multiplicity distributions (upper left panel) resembles the *Z*+jets distribution to a good degree, while the overall rate varies wildly depending on the model point. Note that events containing one jet are produced at almost an identical rate to 2*j* events and comparable to 3*j* events, both in *Z*+jets and all of the benchmark model points we considered. The production rate for different jet multiplicities implies that it could be beneficial to consider DM searches beyond mono-jet, either inclusive, or at fixed jet multiplicities.

Next, the MET (as well as the hardest jet $p_\mathrm{T}$) distributions for mediators of mass $\sim 100 \mathrm{\ GeV}$ with light DM naturally resemble the *Z*+jets distribution in shape, as the kinematics of the MET and hardest jet are determined by the mass scale of the heavy object (i.e. the *Z* boson or the mediator). For heavy mediators, the MET and $p_\mathrm{T}(j_1)$ distributions fall off with a milder slope, suggesting that the signal to background ratio (*S* / *B*) in searches for DM could be improved by requiring a higher $p_\mathrm{T}^\mathrm{min}$ for the hardest jet.

The $p_\mathrm{T}$ distribution of the second hardest jet, on the other hand, seems to display a similar shape in all model points, as well as the *Z*+jets background channel. Requiring a high $p_\mathrm{T}$ on the second jet would hence not improve neither *S* / *B* nor the signal significance, suggesting that in searches which exploit the presence of a second jet, only minimum cuts on $p_\mathrm{T}(j_2)$ should be applied. Note that the modelling of the second jet from LO calculations grossly underestimates the overall rate in case of lighter mediators for $p_\mathrm{T} \gtrsim 100 \mathrm{\ GeV}$, as illustrated in Fig. 6.

## Dark matter production with a top-quark pair

In the spin-0 mediator model, due to the normalisation of the Yukawa couplings in the Lagrangian (11), the most relevant tree-level process at the LHC is

20 $$\begin{aligned} pp\rightarrow X\bar{X} + t\bar{t}. \end{aligned}$$

Such models have in the past been studied in the context of EFT [57–59] and simplified models [46, 48], and searched for at the LHC Run I [60, 61]. Past work on DM interactions with the top quarks has mainly focused on LO estimates, with only a few analyses including NLO corrections. Here we present a comprehensive study of NLO effects of DM interaction with top quarks in the framework of the simplified model. We note that a wide class of so called “top-philic DM” models exist where the LO production is via top loops. Reference [62] studied such a scenario in a minimally model-dependent framework, while more recently, Ref. [44] presented concrete predictions for loop-induced DM production for the current LHC13 run, using the same simulation framework as in this work.

The code and events for the above process can be automatically generated by issuing the following commands in MG5aMC:
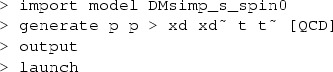


We have checked that our model can reproduce the SM predictions for $pp\rightarrow ht\bar{t}\rightarrow \tau ^+\tau ^-t\bar{t}$ by adjusting the coupling and mass parameters. Note that we use the on-shell renormalisation for the NLO model construction. The top-quark decays can be subsequently performed by MadSpin [63], which keeps production and decay spin correlations.

To illustrate the NLO effects, we consider pure scalar, Eqs. (12) and (13), or pure pseudo-scalar, Eqs. (14) and (15), couplings, and take

21 $$\begin{aligned} g_X=1 \quad \mathrm{and}\quad g_\mathrm{SM}=1 \end{aligned}$$

as the default couplings. With these values, the scalar and pseudo-scalar mediator width is $\Gamma _Y/m_Y\sim 0.06$–0.1 for $m_Y>2m_{X},2m_t$, while $\Gamma _Y/m_Y\sim 0.04$ for $m_Y>2m_X$ and $m_Y<2m_t$. The width for scalar is slightly smaller due to the additional $\beta ^2$ factor where $\beta =(1-4m_{X,t}^2/m_Y^2)^{1/2}$.

### Total cross sections

We start by showing total production rates of $pp\rightarrow X\bar{X}+t\bar{t}$, where the top quark is considered stable. Table 3 shows the LO and NLO cross sections (in pb) for the scalar and pseudo-scalar mediator scenarios, where we use $m_t=172$ GeV. The central renormalisation and factorisation scales are set to half the sum of the transverse mass of the top quarks and the missing transverse energy. We also present scale and PDF uncertainties in % as well as *K* factors.

**Fig. 12:**
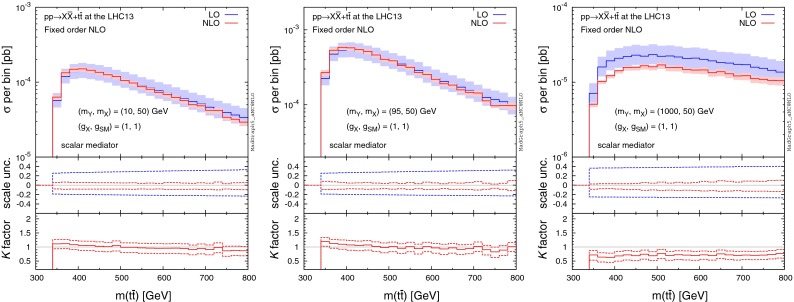
Distribution of the invariant mass of the top-quark pair for $pp\rightarrow X\bar{X}+t\bar{t}$ at the 13-TeV LHC for different mediator masses with the DM mass fixed at 50 GeV, where we assume a pure scalar mediator and Dirac DM. The *middle and bottom panels* show the differential scale uncertainties and *K* factors, respectively

**Fig. 13:**
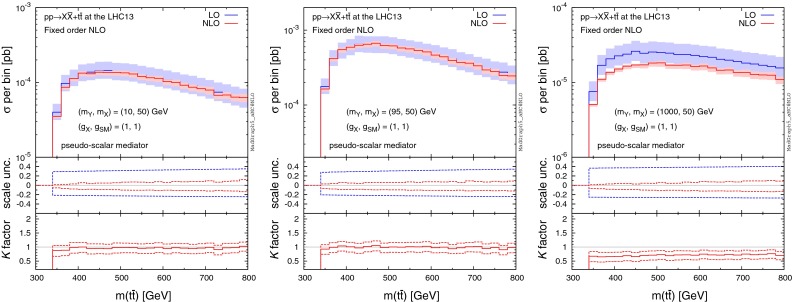
Same as Fig. 12, but for the pseudo-scalar mediator scenario

**Table 3:** LO and NLO cross sections and corresponding *K* factors for DM pair production in association with a top-quark pair for the scalar and pseudo-scalar mediator scenario at the 13-TeV LHC. The uncertainties represent the scale and PDF uncertainties in per cent, respectively. We show several benchmark model points for the mediator and DM masses with the coupling parameters $g_{X}=1$ and $g_\mathrm{SM}=1$

$(m_{Y},m_{X})$ [GeV]			Scalar	Pseudo-scalar
		$\sigma _{\mathrm{LO}}$ [pb]	${2.278\times 10^{1}\,}^{+28.0}_{-20.4}$ ${\pm 4.2\,\%}$	${5.202\times 10^{-1}\,}^{+30.8}_{-22.0}$ ${\pm 6.0\,\%}$
10	Undecayed	$\sigma _{\mathrm{NLO}}$ [pb]	${2.435\times 10^{1}\,}^{+5.4}_{-8.5}$ ${\pm 1.8\,\%}$	${5.431\times 10^{-1}\,}^{+7.4}_{-10.2}$ ${\pm 2.6\,\%}$
		*K* factor	1.07	1.04
		$\sigma _{\mathrm{LO}}$ [pb]	${2.294\times 10^{1}\,}^{+28.0}_{-20.5}$ ${\pm 4.2\,\%}$	${5.500\times 10^{-1}\,}^{+30.8}_{-22.1}$ ${\pm 6.0\,\%}$
(10, 1)	$m_{Y}\!>\!2m_{X}$	$\sigma _{\mathrm{NLO}}$ [pb]	${2.460\times 10^{1}\,}^{+5.4}_{-8.5}$ ${\pm 1.8\,\%}$	${5.739\times 10^{-1}\,}^{+7.4}_{-10.2}$ ${\pm 2.6\,\%}$
		*K* factor	1.07	1.04
		$\sigma _{\mathrm{LO}}$ [pb]	${2.415\times 10^{-3}\,}^{+30.5}_{-21.8}$ ${\pm 5.8\,\%}$	${3.329\times 10^{-3}\,}^{+33.9}_{-23.8}$ ${\pm 8.7\,\%}$
(10, 50)	$m_{Y}\!<\!2m_{X}$	$\sigma _{\mathrm{NLO}}$ [pb]	${2.340\times 10^{-3}\,}^{+5.8}_{-9.1}$ ${\pm 2.8\,\%}$	${3.133\times 10^{-3}\,}^{+7.5}_{-11.0}$ ${\pm 3.9\,\%}$
		*K* factor	0.97	0.94
		$\sigma _{\mathrm{LO}}$ [pb]	${8.226\times 10^{-1}\,}^{+28.7}_{-20.9}$ ${\pm 4.4\,\%}$	${2.442\times 10^{-1}\,}^{+32.2}_{-22.9}$ ${\pm 7.2\,\%}$
100	Undecayed	$\sigma _{\mathrm{NLO}}$ [pb]	${8.391\times 10^{-1}\,}^{+5.3}_{-8.6}$ ${\pm 2.1\,\%}$	${2.431\times 10^{-1}\,}^{+7.6}_{-10.7}$ ${\pm 3.2\,\%}$
		*K* factor	1.02	1.00
		$\sigma _{\mathrm{LO}}$ [pb]	${8.135\times 10^{-1}\,}^{+28.8}_{-20.9}$ ${\pm 4.4\,\%}$	${2.464\times 10^{-1}\,}^{+32.4}_{-23.0}$ ${\pm 7.2\,\%}$
(100, 1)	$m_{Y}\!>\!2m_{X}$	$\sigma _{\mathrm{NLO}}$ [pb]	${8.207\times 10^{-1}\,}^{+4.8}_{-8.3}$ ${\pm 2.1\,\%}$	${2.427\times 10^{-1}\,}^{+7.0}_{-10.4}$ ${\pm 3.2\,\%}$
		*K* factor	1.01	0.98
		$\sigma _{\mathrm{LO}}$ [pb]	${7.986\times 10^{-3}\,}^{+29.5}_{-21.3}$ ${\pm 5.0\,\%}$	${1.404\times 10^{-2}\,}^{+32.9}_{-23.3}$ ${\pm 7.8\,\%}$
(95, 50)	$m_{Y}\!\lesssim \!2m_{X}$	$\sigma _{\mathrm{NLO}}$ [pb]	${7.897\times 10^{-3}\,}^{+5.5}_{-8.8}$ ${\pm 2.4\,\%}$	${1.362\times 10^{-2}\,}^{+7.4}_{-10.8}$ ${\pm 3.5\,\%}$
		*K* factor	0.99	0.97
		$\sigma _{\mathrm{LO}}$ [pb]	${1.571\times 10^{-3}\,}^{+40.2}_{-27.0}$ ${\pm 17.1\,\%}$	${1.827\times 10^{-3}\,}^{+40.4}_{-27.1}$ ${\pm 17.4\,\%}$
1000	Undecayed	$\sigma _{\mathrm{NLO}}$ [pb]	${1.127\times 10^{-3}\,}^{+10.9}_{-13.6}$ ${\pm 8.7\,\%}$	${1.297\times 10^{-3}\,}^{+10.8}_{-13.7}$ ${\pm 8.8\,\%}$
		*K* factor	0.72	0.71
		$\sigma _{\mathrm{LO}}$ [pb]	${7.499\times 10^{-4}\,}^{+40.8}_{-27.2}$ ${\pm 16.8\,\%}$	${8.174\times 10^{-4}\,}^{+40.9}_{-27.3}$ ${\pm 17.0\,\%}$
(1000, 1)	$m_{Y}\!>\!2m_{X}$	$\sigma _{\mathrm{NLO}}$ [pb]	${5.201\times 10^{-4}\,}^{+8.4}_{-12.7}$ ${\pm 8.4\,\%}$	${5.675\times 10^{-4}\,}^{+8.6}_{-12.9}$ ${\pm 8.5\,\%}$
		*K* factor	0.69	0.69
		$\sigma _{\mathrm{LO}}$ [pb]	${7.354\times 10^{-4}\,}^{+40.7}_{-27.2}$ ${\pm 16.8\,\%}$	${8.137\times 10^{-4}\,}^{+41.0}_{-27.3}$ ${\pm 17.0\,\%}$
(1000, 50)	$m_{Y}\!>\!2m_{X}$	$\sigma _{\mathrm{NLO}}$ [pb]	${5.125\times 10^{-4}\,}^{+8.6}_{-12.8}$ ${\pm 8.5\,\%}$	${5.595\times 10^{-4}\,}^{+8.3}_{-12.7}$ ${\pm 8.5\,\%}$
		*K* factor	0.69	0.69

For the total production rates, the NLO effects are very mild for the light mediator case, while they are significant for the heavy case. The inclusion of NLO corrections results in a drastic improvement of the scale uncertainties, from up to 40 % at LO to typically only about 10 % at NLO. Also, the PDF uncertainties are reduced by approximately a factor of two when going from LO to NLO.

Table 3 also shows clear differences between the overall production rates in the cases of scalar and pseudo-scalar mediators. For mediator mass of $\mathcal{O}(10)$ GeV we find that DM production cross section via scalar mediators is an order of magnitude larger compared to the production rate via the pseudo-scalar mediator with the same mass. The large difference occurs due to the fact that in case of $m_X < m_Y \ll m_t$, the production cross section is dominated by the $t \rightarrow t Y_0$ fragmentation. In case of the scalar mediator, the $t \rightarrow t Y_0$ fragmentation function contains terms with soft singularities of the form $(1-x)/x$ – where *x* is the momentum fraction carried by the mediator – causing enhancements in the production rate [64]. The soft-enhanced term is absent in the case of a pseudo-scalar mediator [65], explaining the order of magnitude difference between the total rates of the scalar and pseudo-scalar mediators.

In cases where either DM or the mediator is produced close to threshold, we observe that the production cross section in the pseudo-scalar mediator case is larger. The effect can be attributed to the production rate originating mainly from top fusion diagrams. The production of a DM (Dirac) pair via scalar mediators $t\bar{t} \rightarrow Y_0 \rightarrow X \bar{X}$ at threshold can proceed only via a *P*-wave ($^3P_0$) and is hence suppressed by extra two powers of $\beta = \sqrt{1 - 4m_t^2/s}$ [66]. Conversely, production of DM pair via pseudo-scalar mediators can proceed via an *S*-wave ($^1S_0$) and hence does not suffer any kinematic suppression.

### Differential distributions

For the study of differential distributions, we consider the invariant mass of the top-quark pair ($m(t\bar{t})$), without inclusion of a parton shower. Figures 12 and 13 illustrate the scalar and pseudo-scalar results, respectively, for different mediator masses (off-shell, threshold, and on-shell) with the DM mass fixed at 50 GeV. In all cases the shape of $m(t\bar{t})$ is well modelled by the LO calculation, and including a constant *K* factor reproduces the NLO results to an excellent degree, except in the threshold region. However, the LO calculation suffers from significant scale uncertainties which tend to increase with $m(t\bar{t})$, whereas the scale uncertainties are under much better control at NLO.

**Fig. 14:**
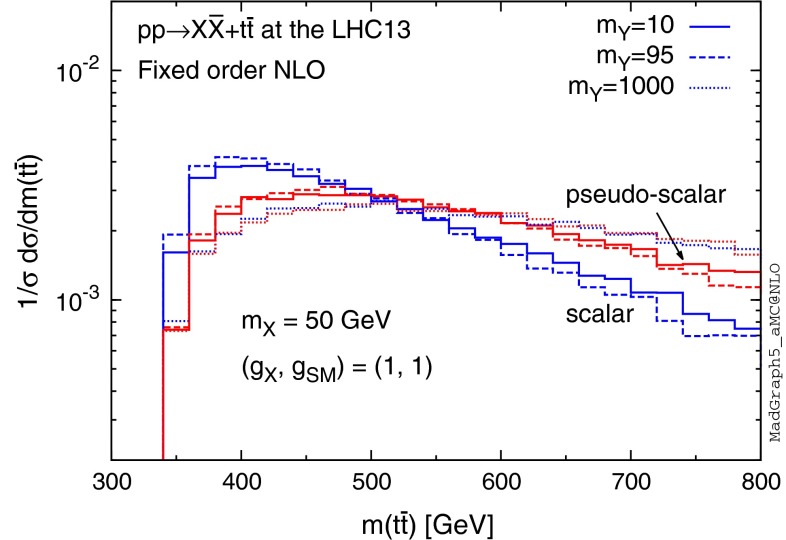
Comparison of $t\bar{t}$ invariant mass distributions between the scalar (*blue*) and pseudo-scalar (*red*) mediator models for different mediator masses

Whether DM is produced via scalar or pseudo-scalar mediators can have a dramatic effect on the shape of the $m(t\bar{t})$ distribution. In Fig. 14 we compare the NLO distributions in Figs. 12 and 13, where we normalise the histograms to unit area to point out the shape differences. We observe that the shape of the distribution is particularly enhanced for $m(t\bar{t}) \gtrsim 500 \mathrm{\ GeV}$ in the case of the pseudo-scalar mediator, while the scalar mediator distribution displays a much more prominent peak at lower $m(t\bar{t})$. The effect is severely damped in case of heavy mediators, where we find no clear differences between the shapes of the scalar and pseudo-scalar mediator distributions. Figure 14 suggests that scalar and pseudo-scalar mediators could be distinguished based on the shape of the $m(t\bar{t})$ distribution, as long as the mediator is sufficiently light and/or does not decay highly off-shell. An analogous observation has been made already in the case of the study of the *CP* properties of the Higgs boson [67].

## Summary

Searches for DM are one of the main endeavours at the LHC Run II. Accurate and precise predictions for production rates and distributions are necessary to obtain robust constraints on DM models and characterise possible DM signals. In this article we have provided a general implementation of the simplified DM model approach into a calculation/simulation framework that allows to systematically evaluate and include NLO QCD corrections to the production of DM at the LHC. We have considered a class of simplified models where DM is a Dirac fermion and couples to the SM via either spin-1 or spin-0 *s*-channel mediators, making no restrictions on chiral couplings. For the purpose of illustration, we analysed the NLO effects on the DM production via vector and axial-vector mediators in the context of mono-jet signals. In addition, we have presented detailed predictions of DM production in association with a top-quark pair via scalar and pseudo-scalar mediators. We presented our results for various DM and mediator masses to cover benchmark points suggested by the ATLAS/CMS DM forum [5].

For MET+jets in the spin-1 mediator model, our results show that higher-order corrections have a significant effect both on the overall production rate as well as on the shape of relevant differential distributions, with a sizeable reduction of the scale and PDF uncertainties. The NLO corrections to the LO production rates can be large, with *K* factors of up to $K\lesssim 2$, and typically occur in parts of the model parameter space where the mass scale of DM and mediator is $\mathcal{O}(10-100)$ GeV. For such scenarios, we also find large NLO effects on the shape of differential distributions in $\mathrm{MET}$ and the transverse momentum of the associated jets. Simplified models with heavy (e.g. $\mathcal{O}(1)$ TeV) mediators/DM do not receive large NLO corrections, and we find that LO predictions describe both total production rates and shapes of differential distributions quite accurately. Distributions of the second hardest jet in the event are well modelled by the parton shower for heavy mediator/DM cases. On the other hand, for mediators/DM with masses of $\mathcal{O}(100)$ GeV, the inclusion of NLO effects is essential for a proper description of $p_\mathrm{T}(j_2)$ and $\eta (j_2)$ distributions, especially in the high-$p_\mathrm{T}$ tails, where the NLO effects can be an order of magnitude.

So-called “giant *K* factors” can occur in NLO computations of DM production rates in the regions where $p_\mathrm{T}^j\gg m_Y$. Such effects can be extremely large when considering mono-jet production rates, especially in phase-space regions with low $\mathrm{MET}$. Imposing a sufficiently large $\mathrm{MET}$ cut and hence avoiding the soft/collinear singularities associated with the mediator emissions from high-$p_\mathrm{T}$ jets efficiently mitigates the effect of giant *K* factors.

In our analysis we have gone beyond FO in perturbation theory and studied the effects of jet multiplicity merging at NLO accuracy. We found that FO calculations model the jet multiplicity and other differential distributions adequately well, with no significant effects on the shapes or overall rates coming from jet sample merging.

Comparisons with the NLO predictions for the leading SM background channel reveal that considerations of either inclusive or exclusive jet samples beyond one jet could be beneficial for increasing the prospects for DM detection. The leading jet $p_\mathrm{T}$ distributions in case of heavy mediators display a milder decrease with the increase in $p_\mathrm{T}$, suggesting that a significant improvement in *S* / *B* could be obtained by focusing on high-$p_\mathrm{T}$ regions. The second hardest jet $p_\mathrm{T}$ distribution, on the other hand, was characterised by a slope similar to the leading backgrounds, implying that more inclusive cuts on the second jet should be used.

For MET+$t\bar{t}$ in the spin-0 mediator model, our results show that the NLO corrections are very mild for the light mediator case, while they are significant for the heavy case. We observed a drastic improvement of the theory uncertainties when going from LO to NLO. We have noted that the shape of the $m(t\bar{t})$ distribution can reveal the chiral structure of the DM–SM interactions, as long as the mediator is relatively light (i.e. ${\lesssim }\mathcal{O}(100)$ GeV).

The DM model we studied in this paper is publicly available in the FeynRules repository [28]. We emphasise that all results presented here have been obtained in the FeynRules/MG5aMC framework, and thus they can be easily reproduced and used in DM searches at the LHC Run II.

## Footnotes

1 A first discussion of the impact of NLO QCD corrections to DM production in a specific simplified model can be found in [17] and more recently in [5, 18].

2 So-called portal models where DM interacts with the Higgs or the $Z/\gamma$ bosons can be included in our framework as well.
